# A focus on recent cases of suicides among Italian children and adolescents and a review of literature

**DOI:** 10.1186/s13052-014-0069-3

**Published:** 2014-07-15

**Authors:** Pietro Ferrara, Francesca Ianniello, Costanza Cutrona, Fabio Quintarelli, Flaminia Vena, Valentina Del Volgo, Olga Caporale, Monica Malamisura, Maria Chiara De Angelis, Antonio Gatto, Antonio Chiaretti, Riccardo Riccardi

**Affiliations:** 1 Institute of Pediatrics, Catholic University of Sacred Heart, Rome, Italy; 2 Campus Bio-Medico University, Rome, Italy

**Keywords:** Suicides, Children, Adolescents, Bullying

## Abstract

Suicidal behaviors are among the leading causes of death worldwide. The large spread of suicide suggests that educational programs and literature to parents or guardians should include information about the potential risks and potential consequences of the onset of the idea of suicide in children. We identified 55 cases of suicide among children and young adults <18-year-old occurring in Italy between 1st January, 2011 and 31st December, 2013. The results point to the need to increase our understanding of the dramatic rise in suicidal behaviors during childhood/adolescence and of the causal pathways linking these behaviors to child-adolescent mental disorders. During routine care visits, pediatricians should be skilled to recognize risk factors for adolescent suicide in order to intervene appropriately.

## Introduction

Suicide is a leading cause of death among children and adolescent around the world and is an important public health problem, in need of attention and intervention [[1]].

According to the last survey of Italian National Institute of Statistics (ISTAT), the first cause of death among children aged between 10–19 years are injuries and poisoning (50.7%), followed by cancers (18.6%) and neurological disease (7.0%) while suicides amount to 6.3%.

In the United States (US), suicide rates doubled in the 15- to 19-year age group and tripled in the 10- to 14-year age group between the 1960s and the 1990s when it began to decrease modestly [[2]]. Moreover, one nationally representative survey in the US found that 12.1% of adolescents experience suicide ideation, 4% develop a suicide plan and 4.1% attempt suicide [[3]].

In England and Wales, between 2001 and 2010, there were 1,523 suicide deaths among those aged 10–19 years (2.25 per 100,000 population; 3.14 for males and 1.30 for female) and suicide rates were higher among those aged 15–19 years (4.04 per 100,000 population) compared with those aged 10–14 years (0.39 per 100,000 population), males approximately 14 times higher and females approximately 6 times higher [[4]].

Compared to other European countries (i.e. Finland and Norway), in which rates are up to five-fold higher, youth suicides are relatively rare in Italy (as in Portugal and Spain) [[5]]. Effectively, in Italy, from 1971 to 2003, a total of 1,871 children and adolescents, aged from 10 to 17 years, committed suicide (about 0.91 per 100,000 population) [[6]].

Therefore it is important to understand the correlates of adolescent suicide to determine whether there are any suicide early warning signs [[2],[3]].

Research reveals that risk factors include family history of suicide or suicide attempts, male gender, parental mental health problems, gay or bisexual orientation, a history of physical or sexual abuse, and a previous suicide attempt [[1]]. Moreover social and environmental risk factors include the presence of firearms in the home, impaired parent–child relationship, living outside of the home (homeless or in a corrections facility or group home), difficulties in school, neither working nor attending school, social isolation, and presence of stressful life events such as legal or romantic difficulties or an argument with a parent [[1]].

ISTAT data about suicide and suicides attempts in adolescents were available up to the year 2010, so the aim of the current study is to update the previous statement of the ISTAT reporting numbers, rates and trends for suicide in children and young adolescents (<14 years) and older adolescents (14–18 years) in Italy between 2011 and 2013 and analyzing epidemiological trends for gender and suicide methods.

The large spread of suicide among adolescents and the lack of information about this problem suggest that educational programs and literature to parents or guardians, in terms of prevention, could be important, in order to provide information about the potential risks and potential consequences of the onset of the idea of suicide in children, who have not yet developed the cognitive faculties to deal with such a serious problem as suicidal ideation on their own.

Moreover, this report will strive to offer a prevention strategy aimed at limiting the phenomenon and trying to detect early risk factors.

## Materials and methods

Our study is based on an internet search for the number of adolescent deaths that resulted from suicide occurring in Italy between 1st January, 2011 and 31st December, 2013. Newspaper indexes, news websites and internet search engines such as Google were used. The search keywords included: suicide, adolescents, risk factors, bullying and abuse. All cases occurred in Italy and young adults aged >18 were excluded. We also looked at the date and time of day when the suicide happened, the underlying cause, the method used, the child’s age and ethnicity.

## Results

We identified 55 cases of suicide among children and young adults <18-year-old. Our small sample size is due to the low prevalence of suicide deaths among Italian young people; our results can be useful to increase the understanding of the phenomenon.

**Figure 1:**
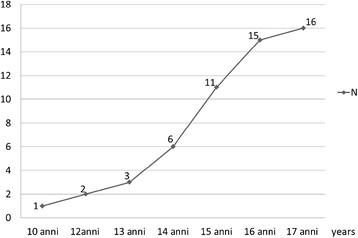
Cumulative number of suicide per age (n. 54 + 1 of unknown age).

Fifteen suicides occurred in 2011, 17 occurred in 2012 and 23 occurred in 2013 (Figure 1). 49/55 (89,1%) were Italian, 2 (3.6%) were Asian, 1 (1.8%) was Romanian, 1 (1.8%) was Latin American, 1 was Slovenian (1.8%) and 1 (1.8%) was African.

Thirty-eight of fifty-five suicides (69.1%) occurred in cities with <100,000 inhabitants and, of these, 50% (19/38) in cities smaller than 25,000, while 17/55 events occurred in cities with >100.000 inhabitants.

We found that suicide rate was higher among boys (58.2%) than girls (41.8%).

The underlying cause was unknown in 26 cases (47.3%); 11/55 (20%) adolescent suicide victims had romantic difficulties, 4 (7.3%) were bullying victims, 3 (5.5%) had been pushed into suicide by an adult, 3 (5.5%) had failed to perform at school, 2 (3.6%) had lost a parent, 3 (5.5%) had a difficult relationship with their parents, 2 (3.6%) were affected by psychiatric disorders and 1 (1.8%) by a serious illness.

The leading suicide method was hanging/suffocation (41.8%), followed by jumping from a height (36.4%). Also firearms (9.1%), jumping in front of a train (5.5%), car accident (3.6%) and self-harm by cutting blades (1.8%) were used. The method was unknown in 1 case (1.8%). Table 1 illustrates the characteristic of children/adolescents and the circumstances surrounding the suicides.

**Table 1:** Characteristic of population and circumstances surrounding the suicides

	**N° (sex)**	**Age**	**Cause**	**Methods**
2011	15 (M/F = 8/7)		5 unknown	9 hanging/suffocation
		1 < 14y	4 romantic difficulties	4 jumping from a height
		13 ≥ 14y	3 difficulties in school	2 jumping in front of an oncoming train
		1 unknown	2 psychiatric disorder	
			1 set on by an adult	
2012	17 (M/F = 12/5)		8 unknown	
			3 romantic difficulties	
		2 < 14y	2 difficult relationship with her parents	6 jumping from a height
		15 ≥ 14y	1 bullying	6 hanging/suffocation
			1 set on by an adult	3 firearm
			1 serious illness/injury	2 car accident
2013	23 (M/F = 12/11)		13 unknown	
			4 romantic difficulties	10 jumping from a height
		3 < 14y	2 bullying	8 hanging/suffocation2 firearm
		20 ≥ 14y	2 loss of the father to death	1 cutting blades
			1 cyberbullying	1 jumping in front of an oncoming train
			1 difficult relationship with her parents	1 unknown

As for the month and the time of day of the events, 3 suicides (5.5%) occurred in January, 2 (3.6%) in February, 6 (10.9%) in March, 4 (7.3%) in April, 8 (14.6%) in May, 7 (12.7%) in June, 2 (3.6%) in July, 7 (12.7%) in August, none (0%) in September, 4 (7.3%) in October, 8 (14.6%) in November, and 4 (7.3%) in December; 13 events (23.6%) occurred in the morning, 14 events (25.5%) in the afternoon, 9 events (16.4%) in the evening, and 7 events (12.7%) during the night. In 12/55 (21.8%) the time of the event was unknown.

## Discussion

Our study was designed to investigate the trends in the prevalence of suicide during the past 3 years (from January 2011 to December 2013) by sex and age, in Italy. We found that there were 55 suicide deaths among young people aged <18 years and that suicide rates were higher among Italians than foreign children/adolescents (8.16 times higher).

Our results also showed that suicides were more common in small towns than in big cities. These area differences in suicide mortality may reflect the aggregated risk of a concentration of people at high risk (compositional effect) and/or the influence of economic, social and cultural aspects of an area on a population’s mental health (contextual effect). Probably in a small country there are a general disadvantage in terms of poor help-seeking behavior, stigmatization of mental health and family problems, and the seasonal fluctuation of employment and social activities. One factor of potential significance could be the inadequate recognition and treatment of adolescents’ problems in outlying areas, due in part to the inherent lack of health services.

Suicide rates were higher among those aged 14–17 years compared with those aged <13 years (males: 5.4 times higher; females: 6.3 times higher).

It has been shown that suicide rates increase with age [[6]]. Data from the US (both public health surveillance in 16 States from 2005 to 2009, and death certificates from 50 States from 2010) showed that the rate of suicide deaths among children aged 10 to 14 years was 1 per 100,000 and for children aged 15 to 19 years was 7 to 8 per 100,000 [[7]]. However, data regarding suicide in children 14 years or younger are not easily available; the World Health Organization provided data on the 5- to 14-year-old only since 1999 but there are nations whose official statistics purposely do not report data on children (Australia is such an example) [[6]].

The most recent data that have been published in Italy show that a total of 91 children and adolescents <18-year-old, died by suicide from 2008 to 2010 [[8]]: 11 children (10 males and 1 female) <13-year-old and 80 children (50 males and 30 females) aged 14–17 years.

Potential explanations include increased access to firearms and potentially lethal drugs, increased rates of psychiatric illness, substance abuse, and other comorbidities, as well as changes in cognitive development. As adolescents develop their capacities for abstract and complex thinking, they are more capable of contemplating life circumstances, envisioning a hopeless future and generating suicide as a possible solution. Younger children who complete suicide are more likely to be of above-average intelligence, possibly exposing them to the developmental level of stress experienced by older children.

Psychosocial factors significantly increase the risk of suicide in children and adolescents, independent of any psychiatric disorders present [[9]]. Common background characteristics of adolescents who die by suicide include broken homes (separation, divorce or death of parents), alcohol or drug misuse and previous self-harm [[10]]. Some of the risk factors include but are not limited to a history of mental disorders, particularly clinical depression, family psychiatric disorder or suicidal behavior, barriers to accessing treatment for mental disorders, unwillingness to seek help because of the public perception attached to mental and substance abuse disorders or to suicidal thoughts [[7],[11],[12]]. Moreover bullying victimization may trigger a sequence of events that results in suicidal behavior [[13]]. In our data, the most frequent known cause of suicide is conflict in a romantic relationship, followed by bullying.

Suicide is more common amongst adolescent males than females in Western societies [[6]]. This indicates that gender differences exist in suicidal behavior across cultures. Public health data from the US on 2010 showed that the rate of suicide deaths among males aged 10 to 19 years was 7 per 100,000 and for females was 2 per 100,000 [[7]].

Risk factors associated with female suicide are less well known but prior research carried out in the Americas has suggested that partner violence and adolescent pregnancy are risk factors [[14]]. Furthermore, females who commit suicide are more likely to have experienced sexual abuse [[14]]. However, the rate of suicidal ideation and suicide attempts are greater in girls than boys. Female high school students are more likely than males to have a specific suicide plan (median of 16% versus 11%, with a range of 13-20% and 8-15% for females versus males, respectively) and one nationally representative survey in the US found that the lifetime prevalence of suicide attempt was greater in adolescent females than males (6% versus 2%) [[3],[15]]. Most studies relate the differences in completion rates to the method chosen. Girls tend to choose less lethal means such as overdose or carbon-monoxide poisoning, whereas boys tend to choose firearms and hanging [[2]]. In our study, 32/55 (58.2%) children are males.

Hanging/suffocation is the most common and lethal method of suicide (41.8% - 23/55), especially for males (46.9% -15/32), followed by jumping from a height (36.4% -20/55), especially for females (56.5% - 13/23).

## Conclusion

Suicide can be prevented. The results point to the need to increase our understanding of the dramatic rise in suicidal behaviors during childhood/adolescence and of the causal pathways linking these behaviors to child-adolescent mental disorders.

Much effort in psychiatric medicine has gone into identifying risk factors for suicide, in order to implement actionable strategies for clinical prediction.

Improved ability to identify individuals who are at risk for suicide attempts and completion may facilitate prevention and enable more appropriate allocation of resources.

Parents can play a major role in facilitating talk about the issue of psychological pain and suicide, but schools should also be a place where parents, teachers and others involved in the health and education of children should build effective suicide preventive strategies for adolescents. Moreover, adolescents who are considering suicide and other violent actions often first confide in peers, therefore students that learn how to recognize peers potentially at-risk for hurting themselves or others and know who to contact in such circumstances may be helpful in preventing a suicide tragedy.

Further monitoring is important to identify changing trends and method of death to improve suicide prevention strategies and this can be achieved by building or using already existing national information systems (such as ISTAT) that collect and analyze suicide data and their associated risk factors (disaggregated by age, sex, ethnicity). In addition, multidisciplinary teams should be created to ensure the most effective response to the health and development of children/adolescents.

During routine care visits, pediatricians should be skilled to recognize risk factors for adolescent suicide, including alcohol and drug misuse, depression, major loss, and recent suicides within a community, in order to intervene appropriately.

To this purpose, adequate training is critical to ensure that pediatricians are prepared to provide effective assessment, prevention and intervention for suicidal behavior.

## Competing interests

The authors declare that they have no competing interests.

## Authors’ contributions

PF conceived of the study, RR and AC participated in its design and coordination and helped to draft the manuscript. FI, CC, FQ and MM contributed in data collection. AG, FV, MCDA, OC and VDV performed review of the literature. All authors read and approved the final manuscript.
